# The Influence of Hydroxyapatite and Alumina Particles on the Mechanical Properties and Corrosion Behavior of Mg-Zn Hybrid Composites for Implants

**DOI:** 10.3390/ma14216246

**Published:** 2021-10-20

**Authors:** Rashid Nazirah, Hussain Zuhailawati, Mohamad Rodzi Siti Nur Hazwani, Tuti Katrina Abdullah, Ismail Azzura, Brij Kumar Dhindaw

**Affiliations:** 1 Biomaterials Niche Area, School of Materials and Mineral Resources Engineering, Engineering Campus, Universiti Sains Malaysia, Nibong Tebal 14300, Penang, Malaysia; nazirahrashid@yahoo.com (R.N.); sitinurhazwani91@gmail.com (M.R.S.N.H.); tutikatrina@usm.my (T.K.A.); 2 Faculty of Mechanical and Manufacturing Engineering, Universiti Tun Hussein Onn Malaysia, Parit Raja 86400, Johor, Malaysia; azzura@uthm.edu.my; 3 Indian Institute of Technology Bhubaneswar, School of Minerals, Metallurgical and Materials Engineering, Khordha 752050, Odisha, India; dhindaw@iitbbs.ac.in

**Keywords:** magnesium alloy, hybrid composite, biodegradable implant, corrosion

## Abstract

Considering the necessity for a biodegradable implant alloy with good biocompatibility and mechanical strength, dual ceramic particles of HAP and Al_2_O_3_ were added to Mg-Zn alloy to produce a new hybrid composite using powder metallurgy. The paper reports the mechanical and corrosion behaviour of Mg-Zn/HAP/Al_2_O_3_ hybrid composites containing variable wt.% HAP and Al_2_O_3_ with 15 wt.% total ceramic content. The powders of Mg, Zn, Al_2_O_3_ and HAP were milled in a high-energy ball mill, and then compacted under 400 MPa and sintered at 300 °C. Density and compression strength increased with increasing Al_2_O_3_ content. HAP facilitated weight gain in Hanks balanced salt solution due to deposition of an apatite layer which promoted anodic behaviour with higher corrosion resistance. A hybrid composite of Mg alloy with 5 wt.% Al_2_O_3_ and 10 wt.% HAP displayed 153 MPa compressive strength, 1.37 mm/year corrosion resistance and bioactivity with a CA:P ratio of 1:1.55 and appears to be the most promising biodegradable implant material tested.

## 1. Introduction

Stainless steel (SS), Co-Cr-Mo, and titanium (Ti) alloys are traditionally used as biomedical alloys owing to their excellent mechanical properties [1], which enable them to play a crucial role in load-bearing implants for the replacement or repair of damaged bones. But they are not bio-degradable and their elastic modulus is higher than that of bone, causing stress shielding and bone absorption. They also release particles or toxic ions into the human body, which causes chronic inflammation, reducing biocompatibility, or tissue loss. In addition, the significant incompatibility of natural bone and implant material may lead to stress, lowering implant stability [2]. A biodegradable implant that decomposes in the body can solve these problems as it will not cause long-lasting physical irritation and furthermore avoids the second surgery to remove the implants [3].

Magnesium (Mg) and its alloys have become attractive candidates for a temporary implant material that avoids the necessity for a secondary operation to eliminate the implant material during healing. They are biodegradable, inherently biocompatible and possess low density and high mechanical properties [4]. Moreover, the modulus of elasticity of magnesium alloy is about 40–45 GPa, which is closer to human bone compared to stainless steel and titanium alloys. Mg also is crucial for health, safe and can be excreted by the kidney [1,5].

However, the main drawback of magnesium and its alloy is their fast degradation rate or corrosion rate in the physiological environment. The fast degradation of magnesium alloy not only results in the implant losing its mechanical integrity before the injured tissues have sufficient time to heal but also leads to serious hydrogen evolution and alkalization of body fluids [6]. Therefore, in order to use Mg as an effective implant, the corrosion rate of Mg needed to be slowed down by alloying the magnesium with non-toxic elements such as Ca, Zn or Zr [7] to form novel magnesium alloys. In this work, Zn was used as an alloying ingredients because Zn can enhance the mechanical and corrosion properties of Mg [7].

One possible way to improve Mg’s mechanical properties and biocompatibility would be to reinforce the magnesium alloy with bioceramic. Calcium phosphate ceramic has been widely used for hard tissue replacements. One type of bioceramic, HAP (Ca_10_(PO_4_)_6_(OH)_2_ [8] has been widely used due its outstanding biocompatibility, good bonding with bone tissues and also its chemical composition, which is very closely similar to the biological apatite existing in human hard tissues. HAP also shows very low solubility as related to beta tricalcium phosphate (β-TCP) in body fluid [9] and, of the phosphate groups, it alone has the feature of biocompatibility. HAP can support bone growth and joints in bone structure without dissolving or breaking down. However, the successful application of HAP ceramic in load-bearing areas is limited because HAP does not have good mechanical strength and has low bioactive property due to its low resorbability [10].

Witte et al. [11] reported the potential of AZ91D magnesium matrix composite reinforced with HAP particles for load-bearing applications. However, HAP is brittle by nature, resulting in a drop in mechanical properties, as reported by Khalil et al. [12] for Mg-HAP and by Soon et al. [13] and Salleh et al. [14] for Mg-Zn/HAP. On the other hand, alumina (Al_2_O_3_) bioceramics have been used widely as components in orthopaedics and dental application since the 1990s. They have been used in dental fixtures and aesthetics (dental restoration) owing to their outstanding biocompatibility and mechanical properties. Furthermore, Al_2_O_3_ is also known to be an excellent bioinert ceramic [15] and has therefore become one of the most widely recommend reinforcement materials for HAP bioceramics [14]. Alumina is a hard ceramic material with high elastic modulus, strong ionic bond and oxygen-rich stoichiometry, which makes it chemically bioinert and stable in the human body [16].

Considering the needs of non-toxic and biodegradable alloy with bioactivity and good mechanical strength, this work investigates a Mg-Zn hybrid composite containing dual ceramic particles of HAP and Al_2_O_3_. Specifically, this paper reports the effect of HAP and Al_2_O_3_ content in varying ratios in Mg-Zn composite on the microstructure, mechanical properties and corrosion behaviour in simulated body fluid (SBF).

## 2. Materials and Methods

Mg contains 98.5% purity (Merck, Darmstadt, Germany), meanwhile Zn is more than 99.9% pure (Alfa Aesar, Lancashire, England), and HAP (Sigma-Aldrich, Burlington, VT, USA) and Al_2_O_3_ (Fluka, Sigma-Aldrich, Burlington, VT, USA) are more than 90.0% pure, respectively. The particle size of Mg, Zn, HAP and Al_2_O_3_ powder is 288.74, 97.64, 2.87 and 10.44 µm respectively. Mg-Zn/HAP/Al_2_O_3_ composites were produced with HAP and Al_2_O_3_ content of 0, 5, 10 and 15 wt.% and 15, 10, 5 and 0 wt.%, respectively, for a total of 15 wt.% ceramic content. Mg and Zn content in the binary alloy matrix was 94 wt.% and 6 wt.%, respectively. Powders were mixed and milled in a Fritch planetary ball mill at 220 rpm for 2 h. The milled and homogenous powders were uniaxially cold pressed using 400 MPa for 2 min and sintered at 300 °C under argon gas flow at 10 °C/min for 1 h.

The sample of sintered composite was observed under SUPRA 35VP field emission scanning electron microscopy (FESEM) using backscattered and secondary electron (BE and SE) modes. Energy-dispersive X-ray (EDX) was used for compositional analysis. Phase identification on the sintered sample was performed using X-ray diffraction (XRD).

The density of the sintered sample was measured and tested using Archimedes’ principle. The samples were immersed in water placed on the Sartorius electronic analytical balance by using four decimals of accuracy. Five readings were recorded and taken for each sample, and the average density was calculated. Meanwhile, for microhardness measurements, Vickers indenter at a load of 300 gf and dwell time of 10 s were conducted on the sample composites. Ten readings were calculated from each sample. A compression test was accomplished at room temperature using the ASTM E9-89a standard test methods.

Most bioactive materials tend to bond to living bone through the formation of an apatite layer that forms on the surface. However, in this study, Hanks Balanced Salt Solution (HBSS) was chosen due to its commercial availability and its continuing use in biomimetic experiments. It is useful to observe the apatite-forming ability on a material’s surface in HBSS to predict the in vivo bonding ability of the material. The mineralization of the apatite layer on the composite was followed according to ASTM-G31-72 [17] in Hanks solution [18]. For the immersion of samples in HBSS, the samples were then ground using SiC emery paper starting from 360 to 2000 grit, and then the samples were polished using 1, 0.3 and 0.05 µm alumina paste. The polished specimens were put under warm airflow for a few minutes. Samples with diameter of 10 mm and thickness of 4 mm were measured and balanced using a four-decimal electronic analytical balance (Sartorius) before the samples were immersed in the HBSS. Before immersion, the volume of HBSS was calculated according to Equation (1):

V_s_ = S_a_/10 (1)

where V_s_ is the volume of HBSS in (mL) necessary to be put into the falcon tube, while S_a_ refers to the surface area (mm^2^) of pellets. Meanwhile, the sintered samples were in the cylindrical disc shape, and S_a_ was calculated according to Equation (2):

S_a_ = 2πr^2^ + 2πrt (2)

where r (radius) and t (thickness) were both in mm unit of the sample.

The total amount of the HBSS calculated as per Equation (1) was then poured into falcon tube, and the samples were submerged at the bottom of a tube and then were put into a water bath heated at 37 °C. After 2 h of immersion, the samples were taken from the water bath heated and were removed from the solution of HBSS. Then, the samples were put in a container and cleaned with dilute chromic acid (a mixture of CrO_3_ and AgNO_3_). The function of dilute chromic acid is to remove the corrosion layer by dissolving Mg(OH)_2_ then rinsing with ethanol, followed by rinsing with de-ionized water. The samples then were put in an oven for 24 h at 70 °C. An average of five measurements was taken for each composition. Finally, the weight of the dried samples (*m_f_*) after the corrosion test was measured, and a percentage change in mass at different times was calculated using the weight loss (*W_L_*) Equation (3) for an in vivo bioactivity test.

(3) $$W_{L}=m_{o}-m_{f}\times 100\%$$

where *m_o_* is the sample weight before the immersion test.

The corrosion rates were calculated by the weight loss according to the following Equation (4) [19]:

(4)  CR=ΔW/(A×t)

where ΔW is the weight loss in gram (g); *A* is the sample area exposed to the solution in centimetre (cm^2^), and *t* is the exposure time in hour (h).

A potentiodynamic polarization test was carried out using an Autolab (PGSTAT-302N) device. A standard three-electrode system was employed, comprising a specimen as working electrode, a platinum plate as a counter electrode and a saturated calomel electrode (SCE) as a reference electrode. Linear polarization tests were done with a scanning rate of 0.5 mv/s and were repeated five times for each composite. The corrosion current densities and the corrosion potentials were directly derived from the linear polarization plots by Tafels extrapolation in which the *E_corr_* and *I_corr_* were acquired from the intersection of the extrapolated *I_red_* and *I_ox_* Tafel lines.

## 3. Results and Discussion

### 3.1. XRD Analysis

**Figure 1:**
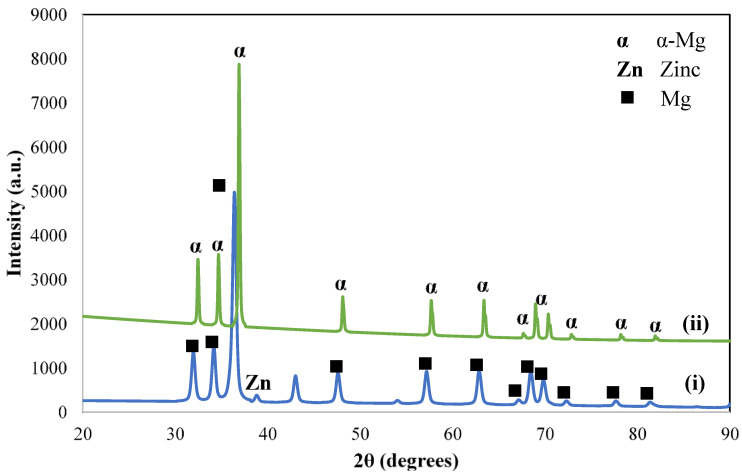
XRD patterns of (**i**) as-milled and (**ii**) sintered Mg-Zn.

Based on Figure 1, the XRD pattern of Mg-Zn alloy shows the absence of Zn peaks in the diffractogram. In the XRD pattern of as-milled Mg-Zn, Zn peaks were still observed in the diffraction angles of 38.90°, 43.09° and 54.22°. As the compact was sintered at 300 °C, the Zn peaks disappeared, and the diffraction angles of sintered Mg-Zn were shifted to the higher diffraction angles, indicating the expansion of the Mg lattice as the Zn was being completely solid-solved into the lattice in sintering process.

**Figure 2:**
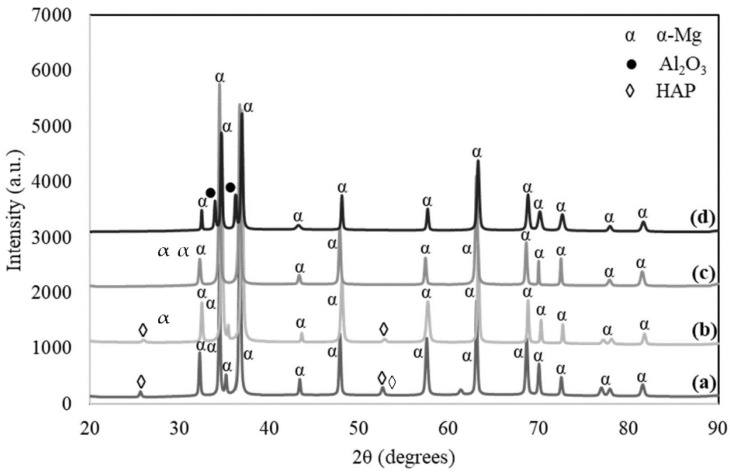
XRD pattern of Mg-Zn/HAP/Al_2_O_3_ composites with different amount of HAP and Al_2_O_3_ (**a**) 0 wt.% Al_2_O_3_, (**b**) 5 wt.% Al_2_O_3_, (**c**) 10 wt.% Al_2_O_3_ and (**d**) 15 wt.% Al_2_O_3_.

Figure 2 displays the XRD diffraction patterns for sintered samples with variable contents of HAP and Al_2_O_3_ at 0, 5, 10 and 15 wt.% Al_2_O_3_ in the Mg-Zn/HAP/Al_2_O_3_ composite. The sharp peaks correspond to the presence of α-Mg and Al_2_O_3_ phase. Zn was not detected, suggesting that the milling process promoting the Zn dissolved into the Mg lattice structure, forming a homogenous solid solution of the α-Mg phase.

### 3.2. Microstructure Analysis

**Figure 3:**
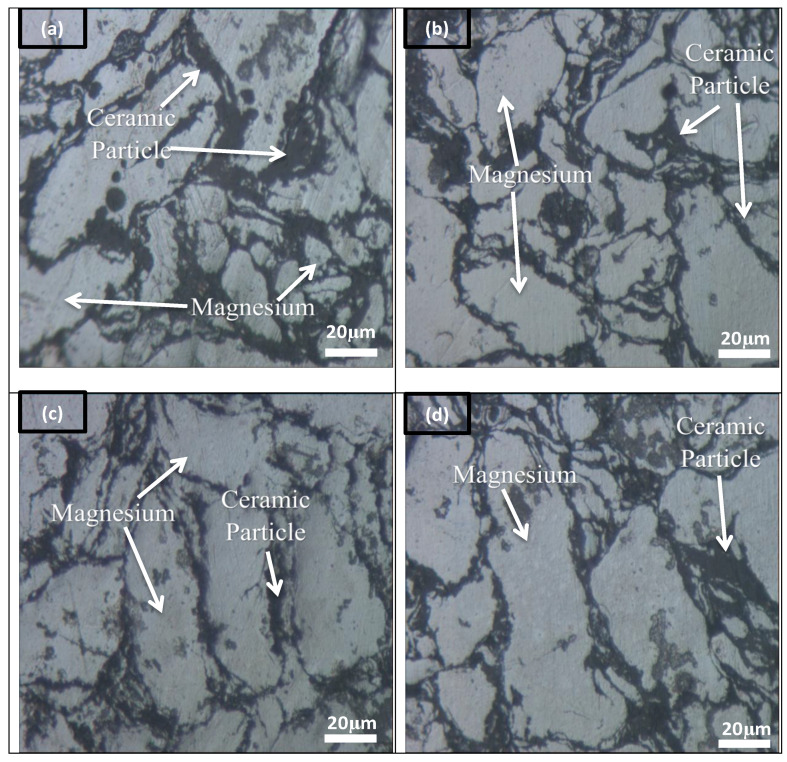
Microstructure of optical micrograph for Mg-Zn/HAP/Al_2_O_3_ with (**a**) 0 wt.% (**b**) 5 wt.% (**c**) 10 wt.% and (**d**) 15 wt.% Al_2_O_3_.

Figure 3 displays optical microstructure images for Mg-Zn/HAP/Al_2_O_3_ with varying amounts of Al_2_O_3_ from 0, 5, 10 and 15 wt.%_._ The Mg matrix is represented by the grey region, while a dark spot, in particular at the grain boundaries, indicates the presence of HAP and Al_2_O_3_ phases or a combination of the two. Table 1 presents the average grain size of the Mg matrix. The grain size changed considerably for composites consisting of 0 wt.% and 5 wt.% Al_2_O_3_ (180.03 µm and 368.40 µm, respectively). However, the grain size developed significantly larger with the addition of 10 and 15 wt.% Al_2_O_3_. Furthermore, the HAP and Al_2_O_3_ particles are located typically at the grain boundaries of the matrix. The presence of ceramic particles at the grain boundary appears to decrease with increasing presence of Al_2_O_3_ particles. Both situations can be explained by the large difference in the particle size of the HAP and Al_2_O_3_ powders, i.e., 2.87 µm and 10.44 µm, respectively. Composites with high HAP content resulted in the refinement of the Mg grain size as HAP is finer, so more particles of HAP are available to resist Mg grain growth by the pinning of boundaries. Thus, composites with 0 and 5 wt.% Al_2_O_3_, which 15 and 10 wt.% HAP, respectively, have a finer Mg grain.

**Figure 4:**
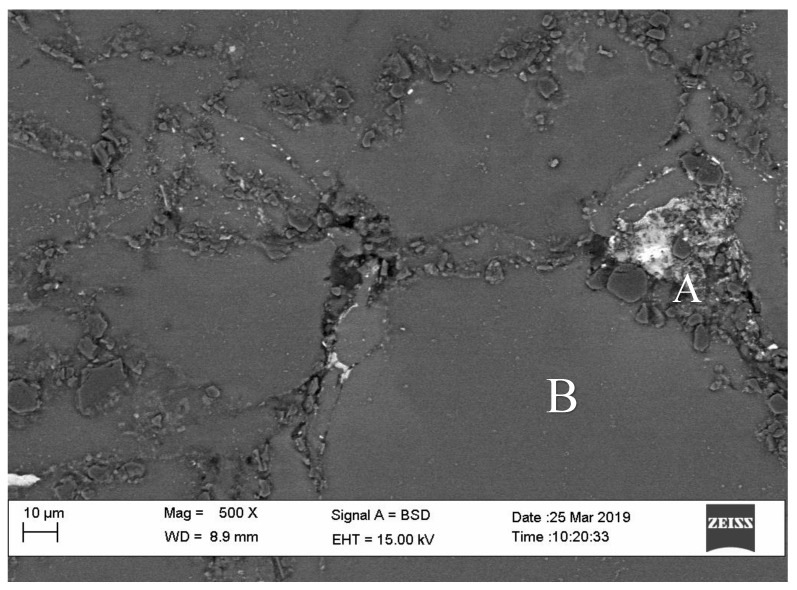
SEM micrograph for overall microstructure of 10 wt.% Al_2_O_3._ Points **A** and **B** are for EDX point analysis.

SEM for all composites is shown in Figure 4 for overall microstructure uniformity, especially the dispersion of the ceramic particles, either HAP or Al_2_O_3_, in the microstructure. Results of EDX analysis on different points for 10 wt.% Al_2_O_3_ containing the composite shows the composition at the grain boundary and in the grain. Point A consist of 12.12 wt.% Al, 8.31 wt.% Ca, 4.43 wt.% P, O, 23.22 wt.%, 1.03 wt.% Zn and 50.89 wt.% Mg, while point B shows high amount of Mg (76.03 wt.%) with trace of 6.48 wt.% Al, 3.16 wt.% Ca, 1.66 wt.% P, and 11.44 wt.% O. These EDX analysis confirm the natural agglomerates of particles at grain boundaries.

### 3.3. Mechanical Properties

Table 2 presents the density and porosity of Mg-Zn/HAP/Al_2_O_3_ composites with various Al_2_O_3_ contents. Addition of Al_2_O_3_ powder resulted in a slight increment in density ranging from 1.846 g/cm^3^ to 1.947 g/cm^3^. This finding suggests that the higher density Al_2_O_3_ particles ensure that Al_2_O_3_ plays a key role in controlling the density of the composites. The density of Al_2_O_3_ is 3.97 g/cm^3^ while the density of pure Mg is 1.736 g/cm^3^. Thus, adding more Al_2_O_3_ caused in a rise in the density of the composite in accordance with the trend of theoretical density estimated by the composite rule of mixtures (ROM). The increase in relative density with increasing Al_2_O_3_ content may also be due to the coarser Al_2_O_3_ particles better supporting the densification of the composite during powder pressing and sintering than did the fine HAP particles. Therefore, composites with 15 wt.% Al_2_O_3_ showed the lowest porosity among the various compositions.

**Figure 5:**
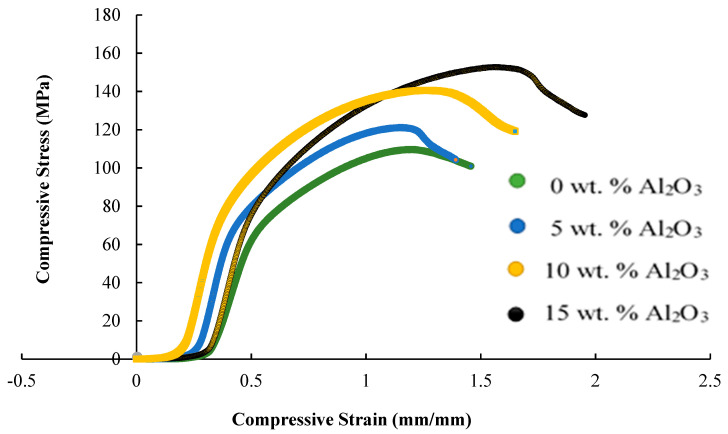
Stress–strain curve for representative samples with different composition of alumina.

**Figure 6:**
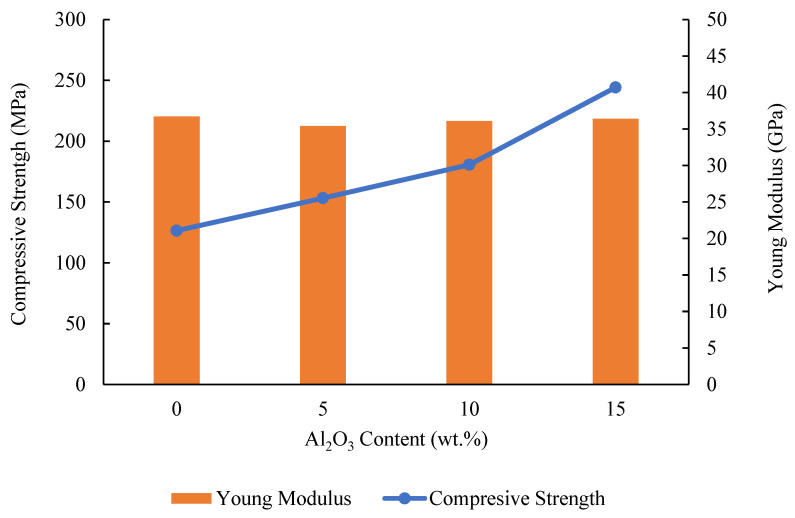
Compressive strength and Young modulus of Mg-Zn/HAP/Al_2_O_3_ composite with different amounts of HAP and Al_2_O_3_.

Stress–strain curves for representative samples with different composition of alumina are displayed in Figure 5, from which compressive strength and Young’s modulus are derived. Average compressive strength of composites with variation in Al_2_O_3_ and HAP content is shown in Figure 6, which suggests that the compressive strength of the Mg hybrid composite can be improved by replacing HAP particles with Al_2_O_3_ hard ceramic particles. The average ultimate compressive strength of the composite increased from 126.48 MPa to 244.20 MPa suggesting that Al_2_O_3_ plays an important role in influencing the strength of the soft magnesium-based composite by hindering the deformation of the Mg alloy matrix. Since Al_2_O_3_ particles in the Mg-Zn/Al_2_O_3_/HAP hybrid composites are hard and brittle, these Al_2_O_3_ particles act as another phase in the matrix, and the movement of dislocations can be restricted [20]. Young’s modulus obtained for all composite was between 35–36 GPa is lower than pure Mg (40–45 GPa), and the values was much closer to human bone (3–20 GPa). Apparently, the compressive strength of the composites was in the range of 126.48 to 244.80 MPa, which fall near the compressive strength of cortical bone (88–230 MPa) and is higher than cancellous bone (0.2–80 MPa), as reported by Pinc et al. [21].

### 3.4. Corrosion Behaviour

#### 3.4.1. Immersion Test

**Figure 7:**
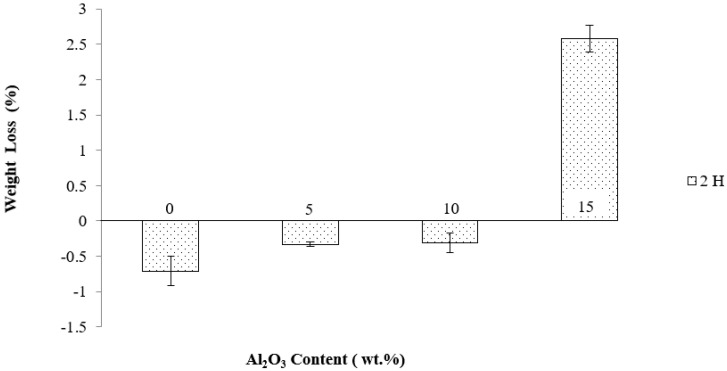
Weight loss by immersion test for Mg-Zn/HAP/Al_2_O_3_ composites different amount of HAP and Al_2_O_3_ after 2 h immersion in HBSS solution.

Figure 7 presents the change in the weight loss of Mg-Zn/HAP/Al_2_O_3_ composites in HBSS after 2 h immersion time. Composites with 10 wt.% Al_2_O_3_ and less gained in weight, while composites with 15 wt.% Al_2_O_3_ lost weight. The amount of weight loss was influenced significantly by the addition of Al_2_O_3_, particularly at 15 wt.% Al_2_O_3_. Increasing Al_2_O_3_ content increased weight loss.

Weight loss due to Mg-Zn matrix degradation is possible as Mg has low resistance to corrosion [22]. However, since the immersion test was conducted in HBSS, the HAP in the composites appear to have encouraged the deposition of the calcium phosphate layer that caused a weight gain of the composites. It is believed that the weight gains for 0, 5 and 10 wt.% Al_2_O_3_ with composites was due to the presence of multiple protective layers contributed from thin Mg(OH)_2_ and the thick apatite layer that was promoted by the presence of HAP in the composites.

On the other hand, composites with 15 wt.% Al_2_O_3_ without HAP showed the highest weight loss at 2.582%. The presence of high Al_2_O_3_ content in the absence of HAP in the Mg matrix did not lead to the creation of an apatite layer at the surface because the Al_2_O_3_ is bioinert, inhibiting the development of an apatite layer. As a bioinert material, Al_2_O_3_ can only remain stable under biological conditions and does not substantially chemically react in the body or exchange electrons with any material it comes in contact with [6]. The formation and creation of an only-Mg(OH)_2_ layer on the surface of the 15 wt.% Al_2_O_3_ composite could not be effective in protecting the magnesium alloy matrix from corrosion.

**Figure 8:**
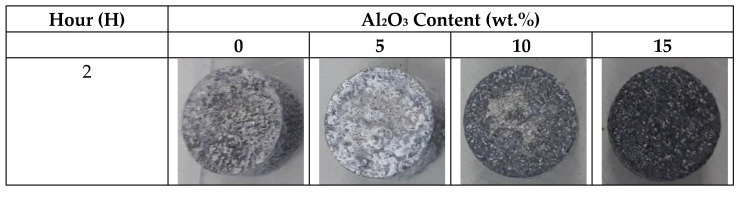
Images of corrode Mg-Zn/HAP/Al_2_O_3_ after an immersion test of 2 and 4 h in HBSS solution.

Figure 8 presents the macroscopic presence of the corroded surface after immersion in HBSS, with its surface degraded owing to the attack of Cl^−^ ions in HBSS solution. Localized pitting was observed for in the samples, covering the entire surface during the course of immersion. The Mg(OH)_2_ layer and possibly the white calcium phosphate layer known as the apatite layer were deposited on the surface of all composites. Results of this research are in agreement with Zhang et al. [22] and Bakhsheshi-Rad et al. [23], who discovered both MgOH_2_ and apatite layers in Mg-Zn alloy and Mg-Ca-Zn, correspondingly.

In general, this observation is consistent with weight loss measurement data wherein a higher content of Al_2_O_3_ was shown to increase the tendency to weight loss owing to the dissolution of the Mg-Zn alloy matrix. The super-protective Mg(OH)_2_ layer and the white calcium phosphate in the protective layer inhibited excessive degradation in composites with HAP. Therefore, we conclude that composites with alumina alone could not prevent the surface from corroding in high Cl^−^ ions medium because Al_2_O_3_ barely interacted with HBSS solution to form calcium phosphate.

**Figure 9:**
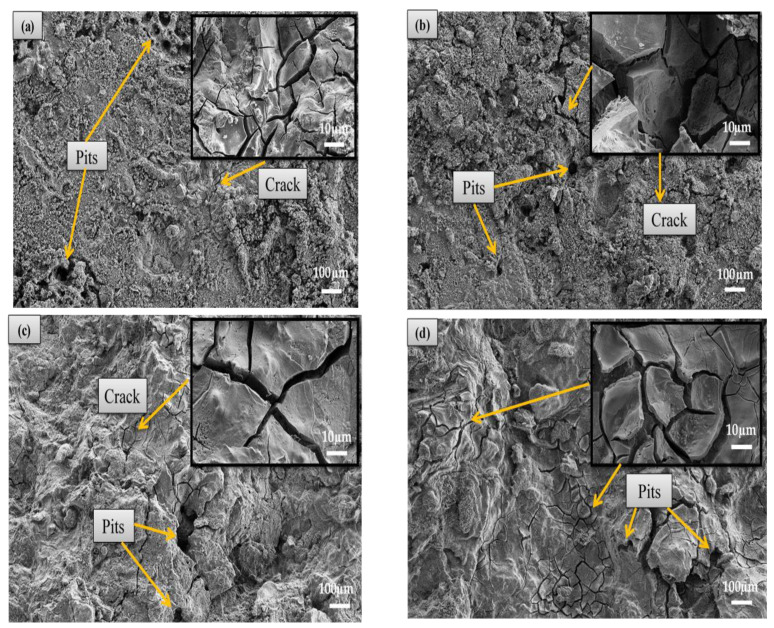
SEM Image Mg-Zn/HAP/ Al_2_O_3_ for 2 h immersed in HBSS (**a**) 0 wt.% (**b**) 5 wt.% (**c**) 10 wt.% and (**d**) 15 wt.% Al_2_O_3_.

Figure 9 shows the morphology of composites under SEM after immersion in HBSS with insert images at higher magnification. The corrosion product was formed on the surface, and the deposited layer covering the samples exhibited a network of cracks and pits with different depths and sizes. The pits and cracks were observed in all composites, but the size and formation of cracks increased with the addition of high Al_2_O_3_ content. The cracks increased the contact area between the corrosive solutions, thus accelerating the corrosion of the alloy. The crack creation has been attributed to the water loss of the surface shrinkage and corrosion products surface shrinkage, as proposed by Bakhsheshi-Rad et al. [23]. The SEM images are in good agreement with this proposal (result of weight loss), where higher Al_2_O_3_ content (at the expense of HAP content) contributed to higher weight loss, while lower Al_2_O_3_ content (associated with higher HAP content) contributed to weight gain.

**Figure 10:**
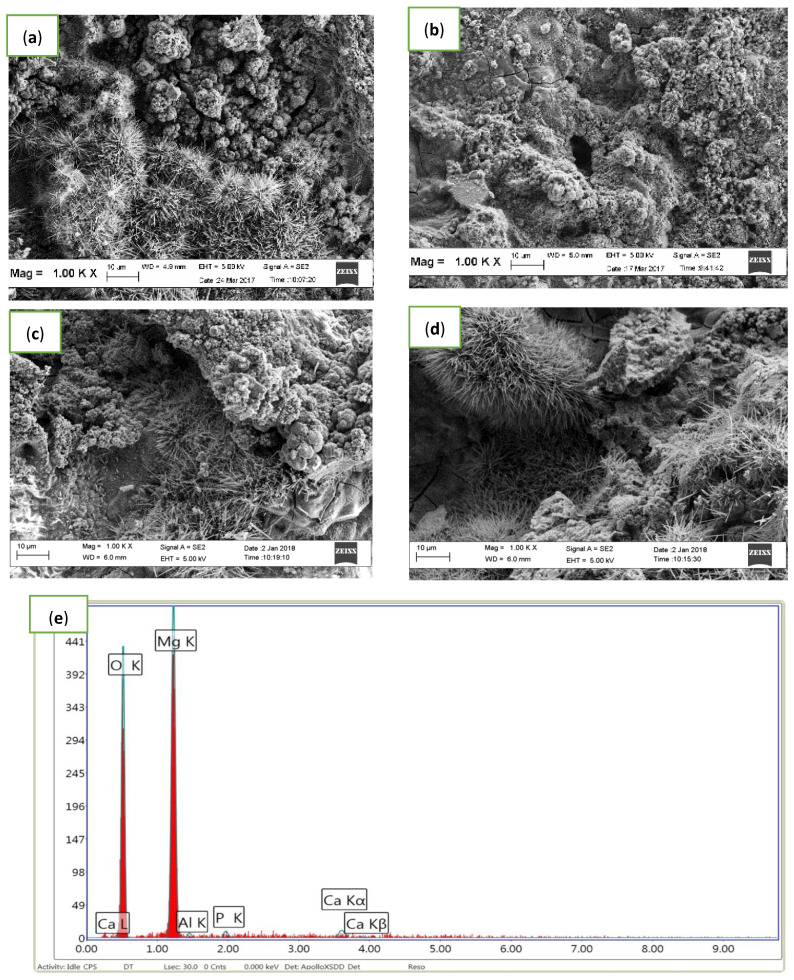
Microstructure of Mg-Zn/HAP Al_2_O_3_ after an immersion test for 2 h in HBSS (**a**) 0 wt.% (**b**) 5 wt.% (**c**) 10 wt.% and (**d**) 15 wt.% Al_2_O_3_ with (e) EDX analysis for 10 wt.% Al_2_O_3_

EDX analysis was used to examine the composition of the deposited layer or corrosion product on the composites. Figure 10 and Table 3 show the presence of magnesium (Mg) and oxygen (O), as well as a small amount of phosphorus (P), calcium (Ca) and aluminium (Al) as the constituents of the corrosion products. The presence of Mg and O in the corrosion products demonstrated the possibility of the formation of magnesium hydroxide Mg(OH)_2_, which acted as the protective barrier against further degradation in the aggressive corrosion medium. The EDX analysis of the 0 wt.% Al_2_O_3_ (with 15 wt.% HAP) indicated the presence of Ca and P functioning as a sponge-like deposit that was found to cover almost all regions on the sample surface with the amount and Ca:P ratio at the highest value of all the composites. This analysis proposes that calcium-deficient HAP was placed on the substrate, as also discovered by [13,14,15,16,17,18,19,20,21,22,23,24,25,26,27,28,29,30,31,32]. EDX reveals that for 0 wt.% Al_2_O_3_, the Ca/P molar ratio was 2.32, which suggests better bioactivity than the other composites.

#### 3.4.2. Polarization Test

**Figure 11:**
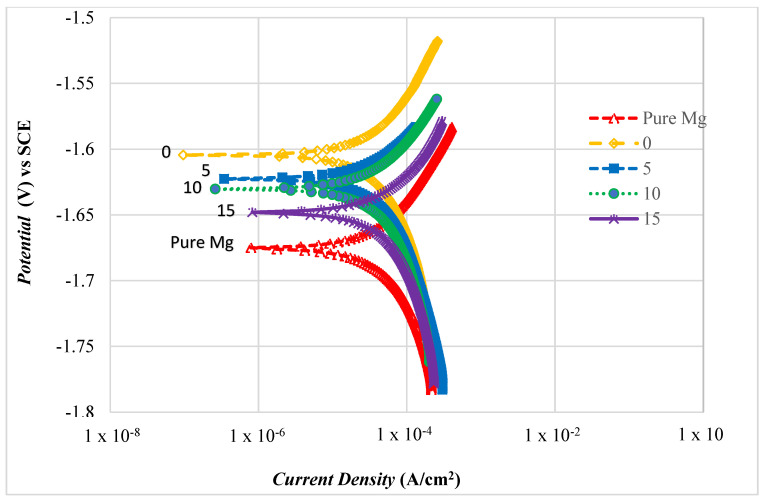
Polarization test for the Mg-Zn/HAP/Al_2_O_3_ composite.

Figure 11 shows the Tafel plot for Mg-Zn/HAP/Al_2_O_3_ composites with different compositions of Al_2_O_3_. In overall, the anodic polarization curves have been attributed to the dissolution of Mg, leading to the formation of Mg^2+^, while the cathodic polarization curves have been associated with the reduction of water [24]. Data derived from the intersection of the anodic and cathodic Tafel line extrapolations curves are corrosion potential (*E_corr_*) and corrosion current (*I_corr_*) and corrosion rate, as summarized in Table 4. Typically, in a potentiodynamic curve, the more positive *E_corr_* and the lower *I_corr_* correspond to lower corrosion rate, or in other words, corrosion resistance is higher. It was found that pure Mg (as a controlled sample) showed the worst corrosion resistance of all the composites with the value of the *E_corr_* (−1.675 V), *I_cor_*_r_ (0.530 × 10^−6^ Acm^−2^) and corrosion rate (3.24 mm/year).

Composites having more positive *E_co_*_rr_ with higher *I_corr_* implies a higher corrosion rate, for two reasons. First, Zn as an alloying element of Mg matrix has a stabilizing effect on the protective film formed on the Mg alloy [22]. It has been informed that Zn can increase the corrosion potential of Mg alloy, thus reducing the corrosion rate [15]. Secondly, the additional ceramic particles with a total amount of 15 wt.% of the total composite composition also can reduced the corrosion rate of the composite.

When coupling the active magnesium with a moderately noble material, galvanic corrosion is the primary prospect that may accelerate corrosion of Mg. Instead, corrosion resistance was found to be improved with the addition of ceramic content into the Mg matrix, indicating that the Mg-Zn/HAP/Al_2_O_3_ composite was the least susceptible to corrosion. Previous work by Ghasali et al. [25] found that the conductivity of the inert materials plays a important role in inhibiting galvanic corrosion between the Al_2_O_3_ and Mg matrix since Al_2_O_3_ is an insulator. In this research, both HAP and Al_2_O_3_ are insulating ceramic particles, which may result in disruptive electric charges because of the influence of an electric field. This means that the electric charged cannot flow freely into the composite or very little electric current will flow through it under the influence of an electric field. Thus, it is expected to have lower *E_corr_*, *I_corr_* and corrosion rate than the magnesium alloy.

*E_corr_*, *I_corr_* and the corrosion rate of the 0 wt.% Al_2_O_3_ composite (with the addition of 15 wt.% HAP) in Table 4 were lower than those of the 15 wt.% Al_2_O_3_ composite (with 0 wt.% HAP). This decrease could be related to the higher HAP content in the 0 wt.% Al_2_O_3_ composite, which known as the hydroxyapatite nuclei, and the hydroxyapatite will grow unexpectedly, consuming the Ca^2+^ and PO_4_^3−^ [22]. Furthermore, the presence in the corrosion products of high amounts of Mg and O in the EDX analysis shows the formation of magnesium hydroxide Mg(OH)_2_, also known as brucite [26]. Brucite forms when the samples are immersed in HBSS solution, and the MgO in the outer layers reacts with the corrosive solution and converts into insoluble Mg(OH)_2_. However, the aggressive Cl^−^ in the HBSS solution transforms Mg(OH)_2_ into a more soluble MgCl_2_ [27]_._ The breakdown of Mg(OH)_2_ decreases the size of the protected area, consequently promoting further dissolution of the sample. The multiple protection effects offered by the corrosion products such as Mg(OH)_2_ and hydroxyapatite at the surface may be the reasons for the slower corrosion rate observed for the 0 wt.% Al_2_O_3_ composite. Witte et al. [28] and Ye et al. [29] also have reported that protective layers on the composite such as Mg(OH)_2_ and HAP composed of multiple corrosion products displayed better corrosion resistance than the single Mg(OH)_2_ layer only.

The more negative value of *E_c_*_orr_ (−1.648 V), and higher values of *I_corr_* (0.474 × 10^−6^ A /cm^2^) and corrosion rate (2.90 mm/year) for the 15 wt.% Al_2_O_3_ containing composite suggests that this composite has poor corrosion resistance. This low corrosion resistance results from the failure to form protective apatite crystals or any form of apatite layer at the surface to prevent the magnesium matrix from corrosion.

However, Al_2_O_3_ is bioinert with poor surface bioactivity, which results in a weak bone-bonding ability and provides no favourable surface for developing biological adherent interfaces with bone [21]. In other words, Al_2_O_3_ is incapable of forming and developing any apatite crystal on its surface under any conditions. Therefore, since the 15 wt.% Al_2_O_3_ composite did not contain HAP, it was unable to initiate other calcium phosphate and apatite when immersed in the HBSS solution. Without HAP particles, the composite had hardly any interaction with the HBSS solution that would allow the formation of calcium phosphate, because Al-OH has no affinity for calcium and phosphate.

Apparently, composites with a lower amount of Al_2_O_3_ shifted the Tafel plot to a more positive value of *E_corr_* and lower value of *I_corr_*, indicating a lower corrosion rate. Thus, composites without Al_2_O_3_ have the best resistance to corrosion in HBSS, as *E_corr_* is the most positive value (−1.604 V) and *I_corr_* is the lowest (0.252 × 10^−6^ A/cm^2^) found in this study. This is attributed to the fact that the composite without any Al_2_O_3_ is the composite with the highest amount of HAP. HAP particles initiated the formation of a thicker protective HAP layer in addition to the Mg(OH)_2_ layer at the composite surface [30], thus increasing the corrosion resistance of the magnesium alloy matrix.

Furthermore, the reduction in the corrosion rate of the composite for 0 and 5 wt.% Al_2_O_3_ can be related to the refinement of the microstructure, which was affected by the addition of HAP and Al_2_O_3_ into the Mg matrix. Thus, it can be concluded that fine grain composites of 0 and 5 wt.% Al_2_O_3_ show better corrosion resistance than do coarse grain composites and hence, have the lowest corrosion rate. This trend is in agreement with other research Waizy et al. [31] and Aung & Zhou [32], which found that the materials with smaller grain size can provide more grain boundaries that can act as a physical barrier that prevents degradation due to corrosion.

## 4. Conclusions

The effect of HAP and Al_2_O_3_ ratio on the physical, mechanical, corrosion and bioactivity properties of magnesium alloy hybrid composite metallurgy was investigated. HAP and Al_2_O_3_ composition was designed from 0 to 15 wt.% and 15 wt.% to 0 wt.% with constant Mg-Zn alloy matrix composition (85 wt.%) to elucidate the properties of new hybrid composites.

Compression strength was increased from 126.48 MPa to 244.20 MPa, respectively with the increase of Al_2_O_3_ content presenting the significant improvement of the strengthening of the soft Mg-Zn alloy provided by the Al_2_O_3_ hard particles. Meanwhile the addition of 15 wt.% HAP (with 0 wt.% Al_2_O_3_) in a polarization test showed that *E_corr_* was shifted to the most positive value, while *I_corr_* and the corrosion rate was lowest. Besides, the highest weight gain was observed, which is correlated to good bioactivity in forming an apatite layer due to the presence of the maximum amount of HAP in this single composite.

Considering the requirement of both high strength and good bioactivity properties, the most suitable composition is achieved by a Mg-Zn alloy matrix hybrid composite consisting of dual ceramic particles of 5 wt.% Al_2_O_3_ and 10 wt.% HAP with 153 MPa compressive strength. The immersion test for corrosion showed that this composition exhibited 0.328% weight gain, 1.37 mm/year corrosion rate and a 1:1.55 Ca:P ratio for bioactivity.

This work highlights the significant of adding dual ceramic particles (bioactive HAP and strong Al_2_O_3_ particles) to a Mg-Zn alloy-based hybrid composite in comparison to a single ceramic addition. Improvement in mechanical, corrosion and bioactivity properties of the new hybrid composite could contribute to the replacement of cortical and cancellous bones with a stronger and bioactive Mg-Zn alloy for biodegradable implant application.

## Figures and Tables

**Table 1:** Average grain size for the Mg-Zn/HAP/Al_2_O_3_ composite.

Al_2_O_3_ Content (wt.%)	Average Grain Size (µm)
0	180.03
5	178.68
10	218.48
15	368.40

**Table 2:** Result for theoretical, relative density and percentage porosity for Mg-Zn/HAP/Al_2_O_3_ composite.

Al_2_O_3_ Content (wt.%)	Theoretical Density (g/cm^3^) (Calculated from ROM)	Sintered Density (g/cm^3^)	Relative Density (Sintered Density Divide Theoretical Density × 100) (%)	Porosity (%)
**0**	1.947	1.846	94.81	5.19
**5**	1.959	1.875	95.71	4.29
**10**	1.971	1.899	96.35	3.65
**15**	1.984	1.947	98.14	1.86

**Table 3:** EDX analysis for Mg-Zn/HAP/Al_2_O_3_ hybrid composite with different amounts of HAP and Al_2_O_3_ after immersion in HBSS solution for 2 h.

Al_2_O_3_ Content (wt.%)	Atomic Percentage (%)					Ratio Ca/P
	Mg	O	Ca	P	Al	
**0**	39.45	59.52	0.72	0.31	0.00	2.32
**5**	39.72	59.08	0.34	0.22	0.63	1.55
**10**	39.50	58.94	0.29	0.40	0.87	0.73
**15**	39.93	58.69	0.16	0.28	0.94	0.57

**Table 4:** Electrochemical profile for the Mg-Zn/HAP/Al_2_O_3_ composite.

Composition Al_2_O_3_ (wt.%)	Corrosion Potential *E_corr_*, vs SCE (V)	Corrosion Current Density *(I_corr_*, A/cm^2^) × 10^−6^	Corrosion Rate (mm/Year)
**Pure Mg**	−1.675	0.530	3.24
**0**	−1.604	0.252	1.25
**5**	−1.622	0.281	1.37
**10**	−1.630	0.379	2.32
**15**	−1.648	0.474	2.90

## Data Availability

Data sharing not applicable.
